# The association between vaccination confidence, vaccination behavior, and willingness to recommend vaccines among Finnish healthcare workers

**DOI:** 10.1371/journal.pone.0224330

**Published:** 2019-10-31

**Authors:** Linda Cecilia Karlsson, Stephan Lewandowsky, Jan Antfolk, Paula Salo, Mikael Lindfelt, Tuula Oksanen, Mika Kivimäki, Anna Soveri

**Affiliations:** 1 Department of Psychology, Åbo Akademi University, Turku, Finland; 2 School of Psychological Science, University of Bristol, Bristol, United Kingdom; 3 School of Psychological Science, University of Western Australia, Perth, Australia; 4 Department of Psychology, University of Turku, Turku, Finland; 5 Finnish Institute of Occupational Health, Turku, Finland; 6 Department of Theological Ethics, Åbo Akademi University, Turku, Finland; 7 Clinicum, Faculty of Medicine, University of Helsinki, Helsinki, Finland; 8 Institute of Clinical Medicine, University of Turku, Turku, Finland; University of Campania, ITALY

## Abstract

Information and assurance from healthcare workers (HCWs) is reported by laypeople as a key factor in their decision to get vaccinated. However, previous research has shown that, as in the general population, hesitancy towards vaccines exists among HCWs as well. Previous studies further suggest that HCWs with a higher confidence in vaccinations and vaccine providers are more willing to take the vaccines themselves and to recommend vaccines to patients. In the present study with 2962 Finnish HCWs (doctors, head nurses, nurses, and practical nurses), we explored the associations between HCWs’ vaccination confidence (perceived benefit and safety of vaccines and trust in health professionals), their decisions to accept vaccines for themselves and their children, and their willingness to recommend vaccines to patients. The results showed that although the majority of HCWs had high confidence in vaccinations, a notable share reported low vaccination confidence. Moreover, in line with previous research, HCWs with higher confidence in the benefits and safety of vaccines were more likely to accept vaccines for their children and themselves, and to recommend vaccines to their patients. Trust in other health professionals was not directly related to vaccination or recommendation behavior. Confidence in the benefits and safety of vaccines was highest among doctors, and increased along with the educational level of the HCWs, suggesting a link between confidence and the degree of medical training. Ensuring high confidence in vaccines among HCWs may be important in maintaining high vaccine uptake in the general population.

## Introduction

The introduction of vaccinations has led to improvements in global health by dramatically decreasing the spread of infectious diseases [1]. Global health organizations, such as the WHO, give high priority to the development and implementation of effective immunization programs. In spite of the indisputable benefits of vaccines, vaccination uptake has decreased in some parts of the world, and some vaccine-preventable diseases are on the increase [2,3]. The question of why some individuals choose to reject vaccines, even though they are safe, easily accessible, and often free of charge or at least affordable, is becoming an increasingly important topic of research.

Several studies have found that a variety of contextual, social, individual, and vaccine-specific factors are associated with accepting, postponing, or refusing vaccination [4–11]. From a psychological perspective, an important correlate of vaccination behavior is vaccination confidence, which refers to attitudes and beliefs related to the benefits and safety of vaccines, as well as trust in vaccine providers, such as healthcare workers (HCWs), health authorities, and policy makers [4]. Systematic reviews show that individuals who perceive vaccines as less beneficial and safe, more often reject scheduled vaccinations for their children and influenza vaccinations for themselves [12–14]. Systematic reviews also suggest that greater trust in HCWs is associated with a higher likelihood of accepting vaccines [12–14]. HCWs play a key role in maintaining and increasing vaccine uptake, as they are involved in the immunization process and communicate with patients about vaccines. Studies based on self-reports from laypeople show that, in general, HCWs are considered to be the most reliable source of vaccine information [8,10,15–17]. Furthermore, advice given by HCWs is the most frequently reported reason for vaccine acceptance among the general public, while lack of recommendation is mentioned as a reason for non-vaccination [8,12,18–20]. HCWs’ recommendation behavior therefore seems to play a pivotal role in vaccination decisions.

Although the vast majority of HCWs endorse vaccination, negative attitudes towards vaccination can be found among HCWs as well [21–25]. According to a recent systematic review, HCWs with lower confidence in the benefits and safety of vaccines are less willing to recommend vaccines to their patients ([21]; see also, [26,27]), and less likely to accept vaccinations for themselves [28,29]. An important aspect to consider when investigating vaccine attitudes in HCWs, is that compared with the general public, HCWs are expected to have acquired evidence-based information on vaccines. The HCWs’ perception about the benefits and safety of vaccines should, thus, not only be considered as a measure of their attitudes to vaccines, but also as an indicator of their vaccine-related knowledge.

Trust in their fellow health professionals is another aspect of vaccination confidence worthy of exploration among HCWs, as they too can experience a lack of trust in the health system, even though they are a part of it themselves [22,24]. However, the association between trust in the health system and vaccination and recommendation behavior has rarely been studied in HCW samples [30]. Učakar et al [31] surveyed 897 Slovenian physicians and found that those who regularly or occasionally take the influenza vaccine, more often reported that they trust professional vaccine recommendations, compared with those who no longer or never vaccinate themselves against influenza. A French study ([27]; see also, [32]) on 2586 general practitioners showed that lower trust in official and scientific sources of vaccine information (such as health authorities, scientific sources, or specialist colleagues) was associated with a reduced likelihood of recommending vaccines. The general practitioners were also less likely to recommend vaccines when they believed that severe adverse effects from vaccines were likely and had doubts about the utility of vaccines. Thus, the results from this study suggested that all three components of vaccination confidence were related to general practitioners’ willingness to recommend vaccines to their patients.

In the present study, we investigated whether the perceived benefit of vaccines, perceived safety of vaccines, and/or the degree of trust in health professionals, were related to whether the HCWs’ accept vaccinations for themselves and for their children, and whether they recommend childhood and influenza vaccines to patients who are hesitant towards vaccines. More specifically, we hypothesized that HCWs who perceive vaccines as less beneficial, less safe, and/or have less trust in health professionals, are 1) more likely to have hesitated, postponed, or rejected a vaccination for their children, 2) more likely to have rejected the influenza vaccine for themselves, and 3) are less likely to recommend childhood and influenza vaccines to vaccine-hesitant patients.

## Methods

### Study context

In Finland, where the present study was carried out, childhood vaccinations are voluntary and free of charge and almost all vaccines are administered by a public health nurse at a child health clinic in accordance with the national vaccination program [33,34]. The influenza vaccines are administered to HCWs at their place of work and are paid for by their employer [35]. Since March 2017, the new Infectious Disease Act 48§5 requires HCWs working with patients belonging to risk populations to be immunized for measles, chickenpox, and influenza. HCWs who do not accept annual influenza vaccinations may be assigned to other tasks [36,37].

### Ethics statement

The project received ethical approval from the ethics committee of the Hospital District of Helsinki and Uusimaa. Before completing the questionnaire, respondents were presented with information about the purpose of the study and management of the data. Participants were asked to indicate their understanding of the information provided and expressed consent by checking a box. The respondents were informed that participation was voluntary and that they could withdraw from the study at any time.

### Study population

We sourced participants from the Finnish Public Sector study, which is a large on-going cohort study among municipal and hospital employees [38]. We sent an invitation to participate in an electronic survey to the 8770 hospital staff members who had participated in the study wave the preceding year (2017). For practical reasons, the survey was sent out in two phases. In the first round (February 28^th^, 2018), the survey was sent to hospital personnel in the regions of Forssa (*n* = 916), Kanta-Häme (*n* = 1201), Pietarsaari (*n* = 761), and Vaasa (*n* = 1321), and in the second round (March, 13^th^, 2018) to hospital personnel in Pirkanmaa (*n* = 4571).

### Measures

The survey questions were developed by the authors of the present study after a literature review and discussions with experts working within a nursing education programme in Finland. The dimensions of the survey relevant for the aim of the present study concerned: 1) beliefs about the benefits of vaccines, 2) beliefs about the safety of vaccines, 3) general trust towards healthcare professionals, 4) own vaccination behavior (i.e., whether respondents had accepted childhood vaccines for their own children and influenza vaccines for themselves), and 5) recommendation behavior in cases where patients are hesitant towards vaccines (S1 Appendix).

The survey questions concerning perceived benefit of vaccines, perceived safety of vaccines, and trust in health professionals, can be seen in Table 1. The questions on the benefits and safety of vaccines were created according to current official vaccination guidelines and evidence-based information [39].

**Table 1:** Survey questions measuring perceived benefits of vaccines, perceived safety of vaccines, and trust in health professionals.

Survey question	Item label	Topic	Knowledge type
Vaccinating healthy children helps to protect others by stopping the spread of disease.	HerdImmunity	Benefit	General
It is better to be immunized trough the disease than through the vaccine.	Immunized^a^	Benefit	General
Children need vaccines for diseases that are not common anymore.	NotCommon	Benefit	General
Childhood vaccines are effective in protecting against disease.	ChildProtection	Benefit	General
Measles is a very serious disease.	ChildSerious	Benefit	Specific
A good hygiene will make measles disappear from society–the vaccine is not necessary.	ChildNecessary^a^	Benefit	Specific
The influenza vaccines are effective in preventing against the disease.	FluProtection	Benefit	Specific
It is not worth getting the influenza vaccine, as the influenza symptoms are not serious.	FluSerious^a^	Benefit	Specific
Good hand hygiene and other preventive efforts are enough for avoiding the influenza even without vaccination.	FluNecessary^a^	Benefit	Specific
Vaccines can cause autism.	Autism^a^	Safety	Specific
Vaccines contain dangerous quantities of mercury.	Mercury^a^	Safety	Specific
The risk of side effects outweighs the protective benefits of the childhood vaccines.	ChildSideEffects^a^	Safety	General
Childhood vaccines are safe.	ChildSafety	Safety	General
The risk of side effects outweighs the protective benefits of the influenza vaccines.	FluSideEffects^a^	Safety	Specific
The influenza vaccines are safe.	FluSafety	Safety	Specific
I think it is good that patients/parents question the doctors’ ability to make correct diagnoses.	QuestionDoctors^a^	Trust	-
When healthcare professionals make medical decisions, they have the patients’ best interest in mind.	PatientsBest	Trust	-
Doctors are too authoritative towards their patients.	DoctorsAuthority^a^	Trust	-
Parents should leave the decisions that concern their children’s health in the healthcare professionals’ hands.	HealthDecisions	Trust	-

The term knowledge type refers to the follow-up analyses concerning profession (see, Results).

^a^Reverse-scored item

#### Perceived benefits of vaccines

Nine statements were created to measure the HCWs’ perceptions and knowledge of the benefits of vaccinations. Items were of varying polarity; e.g., “Childhood vaccines are effective in protecting against disease” vs. “It is not worth getting the influenza vaccine, as the influenza symptoms are not serious”. Some of the questions queried knowledge about vaccines in general, whereas others concerned knowledge about specific vaccines or vaccine-preventable diseases (measles or influenza). The respondents were asked to rate how much they agreed with each statement on a scale from 1 (strongly disagree) to 5 (strongly agree).

#### Perceived safety of vaccines

Six statements were formulated to measure the perceived safety of vaccines (e.g., “Childhood vaccines are safe”, “The risk of side effects outweighs the protective benefits of the influenza vaccines”). As with the questions regarding the perceived benefits of vaccines, some statements were related to vaccines in general, and others to specific vaccines. Two of the specific questions concerned common misinformation about vaccines; that vaccines could cause autism and that vaccines contain dangerous quantities of mercury. Again, the respondents rated how much they agreed with each statement on a scale from 1 (strongly disagree) to 5 (strongly agree).

#### Trust in health professionals

Four statements were created to measure the HCWs’ trust in the intentions and professional competence of doctors and health professionals in general (e.g., “When healthcare professionals make medical decisions, they have the patients’ best interest in mind”). The respondents were asked to rate how much they agreed with each statement on a scale from 1 (strongly disagree) to 5 (strongly agree).

#### Own vaccination behavior

To understand the HCWs’ own vaccination behavior, the survey included 1) three questions on whether or not they had their children vaccinated with the childhood vaccines (“Have you ever hesitated in letting your child(ren) receive any of the childhood vaccines?”, “Have you ever postponed a vaccination for your child(ren) with any of the childhood vaccines?”, and “Have you ever decided not to let your child(ren) receive any of the childhood vaccines?”), and 2) one question probing whether the respondents had taken the influenza vaccine during the preceding influenza season. The questions concerning childhood vaccinations were administered only to respondents who reported having children.

#### Recommendation behavior

Respondents who reported discussing or administering vaccines to patients on a weekly basis were asked two questions about how they recommended vaccines to vaccine-hesitant patients. These questions were “How do you proceed if a parent is unsure about a vaccination decision concerning the childhood vaccines (and the child does not have any medical contraindications)?” and “How do you proceed if a patient is unsure about a vaccination decision concerning an influenza vaccine (and the patient does not have any medical contraindications)?” For each question, the respondents could choose between three response options; “I try to guide the parent/patient towards vaccinating”, “I do not try to guide the parent/patient in any direction”, or “I try to guide the parent/patient towards not vaccinating”.

### Statistical analysis

We analyzed the data using structural equation modeling (SEM), or more specifically, structural regressions (SR). The four outcome measures constituted: 1) childhood vaccination decisions concerning own children, 2) own influenza vaccinations, 3) recommendation behavior concerning childhood vaccines, and 4) recommendation behavior concerning influenza vaccines. The first outcome variable was created based on the three questions regarding the HCWs’ decisions on vaccines for their child(ren) (i.e., whether they had hesitated in a vaccination decision, postponed a vaccination, or rejected a vaccination altogether). The responses on the new variable were coded as an ordered factor where: 0 = no hesitancy, no postponing, and no rejection of a vaccination; 1 = hesitancy, but no postponing or rejection of a vaccination; 2 = postponing, but no rejection of a vaccination; or 3 = rejection of a vaccination. However, if a respondent had indicated that the reason for postponing a vaccination was that the child had been ill at the initial vaccination occasion, their response was coded as 0 (= no hesitancy). The response was coded as no hesitancy also if the reason for hesitating or rejecting a childhood vaccination was allergies or contra-indicative medical conditions. The outcome variable on the HCWs’ own influenza vaccinations was coded as: 0 = had received the vaccine against influenza; or 1 = had not received the vaccine against influenza. If a respondent had indicated that the reason for not receiving the vaccination was allergies or other contra-indicative medical conditions, their response was again coded as 0. The two variables concerning recommendation behavior were coded as ordinal factors where: 0 = try to guide the parent/patient towards vaccinating; 1 = do not try to guide the parent/patient in any direction; and 2 = try to guide the parent/patient towards not vaccinating.

The outcome measures were included in separate models, and, hence, four SR models were fitted to the data. In the initial model specification, each model included an outcome measure as an observed variable that was regressed on three latent factors: 1) perceived benefits of vaccines (Benefit; nine indicators), 2) perceived safety of vaccines (Safety; six indicators), and 3) trust in health professionals (Trust; four indicators; see, Table 1 for indicators specified to load on the factors). Before fitting the full SR models, we evaluated the fit of the measurement part of the models (the latent factors) by confirmatory factor analysis (CFA). The three latent factors were first analyzed in separate CFA models to investigate the unidimensionality of indicators, and then the three-factor model was evaluated.

We conducted the analyses using the lavaan package (version 0.6–3) [40] in R (version 3.5.0) [41]. Due to the ordinal and categorical nature of the response variables, as well as the non-normal distribution of responses, the analyses were conducted using robust WLS (WLSMV) estimation with delta parameterization. The WLSMV estimator analyzes the asymptotic covariance matrix generated from the polychoric correlations between the indicators. Regression coefficients were estimated using the probit link function. The probit regression coefficient represents the change in the standard normal distribution (z-score) of the outcome variable, given a one-unit increase in the predictor. Missing data was handled with pair-wise deletion.

## Results

Altogether, 4286 individuals responded to the survey (response rate 49%). The present study only included those hospital personnel who may work with vaccinations, such as doctors, head nurses, nurses, and practical nurses. Other health professionals (e.g., psychologists and physiotherapists) and other personnel working at hospitals (e.g., within administration and human resource management) were excluded from the sample of respondents. Therefore, the final sample included 2962 HCWs (Table 2) with the mean age of 44.80 years (*SD* = 11.14, range = 20–67).

**Table 2:** Descriptive information on the HCWs (*N* = 2962).

	*N*	*%*
Sex		
Female	2626	88.7
Male	336	11.3
Profession		
Doctors	416	14.0
Head nurses	263	8.9
Nurses	1834	61.9
Practical nurses	449	15.2
Employer		
Forssa^a^	302	10.2
Kanta-Häme^b^	327	11.0
Pietarsaari^c^	306	10.3
Pirkanmaa^d^	1569	53.0
Vaasa^e^	458	15.5

^a^Municipal Authority of Wellbeing in Forssa district.

^b^Hospital District of Kanta-Häme.

^c^Pietarsaari Health and Social Services.

^d^Hospital District of Pirkanmaa.

^e^Hospital District of Vaasa.

Only 0.91% of the total observations on the 19 questions measuring vaccination confidence (benefit, safety, and trust) were missing, while between 0.04% and 2.22% of the responses on the vaccination and recommendation behavior questions were missing (see, S1 Table for the number and percentage of missing responses per variable).

Table 3 presents the HCWs’ responses to the questions concerning perceived benefit and safety of vaccines, and trust in health professionals. In the table, reverse-scored items have been recoded so that all items have the same polarity (e.g., % Pos = positive attitudes towards vaccines or healthcare professionals). In general, the HCWs perceived vaccines to be beneficial and safe, and reported trust in health professionals. However, depending on the item, 1.7%-38.1% of the HCWs did not consider vaccines beneficial, 4.6%-25.7% reported that they did not consider the vaccines safe, and 3.9%-42.5% reported negative attitudes on the measures of trust. Compared with the whole sample, the HCWs who have the right to administer vaccines (doctors, head nurses, nurses), and who reported that they either discuss or administer vaccines on a weekly basis (*n* = 751), considered vaccines more beneficial and safe (see, S2 Table). Between 0.9% and 31.2% of those HCWs responded that they did not perceive vaccines as beneficial, and between 4.0% and 17.9% did not perceive vaccines as safe. Finally, between 1.8% and 38.6% reported low trust in health professionals.

**Table 3:** Descriptive information on the HCWs’ attitudes towards the benefits and safety of vaccines, as well as trust in health professionals.

	Doctors			Head nurses			Nurses			Practical nurses			Total		
	% Neg	% Mid	% Pos	% Neg	% Mid	% Pos	% Neg	% Mid	% Pos	% Neg	% Mid	% Pos	% Neg	% Mid	% Pos
Benefit															
HerdImmunity	1.4	0.2	98.3	3.8	0.4	95.8	2.5	1.0	96.5	4.3	4.7	91.0	2.7	1.4	95.9
Immunized	6.5	5.8	87.7	14.4	15.6	70.0	17.3	13.0	69.7	26.2	19.9	53.9	16.9	13.3	69.8
NotCommon	5.8	6.5	87.7	7.3	5.8	86.9	9.3	9.5	81.2	16.0	13.7	70.3	9.7	9.4	80.9
ChildProtection	1.5	0.0	98.5	3.8	1.9	94.2	2.2	2.1	95.7	4.9	5.2	89.9	2.7	2.3	95.1
ChildSerious	1.5	3.7	94.9	3.9	8.1	88.0	5.4	7.4	87.2	8.2	12.7	79.1	5.1	7.7	87.2
ChildNecessary	1.9	0.7	97.3	1.9	3.1	95.0	1.3	5.2	93.5	3.0	12.0	85.0	1.7	5.4	92.9
FluProtection	14.2	6.6	79.2	23.6	14.3	62.2	42.8	12.4	44.8	49.8	15.1	35.2	38.1	12.2	49.7
FluSerious	3.2	2.2	94.7	6.8	5.3	87.8	6.9	10.2	82.9	11.3	21.3	67.3	7.0	10.3	82.7
FluNecessary	10.2	3.1	86.7	13.4	3.4	83.2	26.0	8.4	65.6	36.4	14.1	49.6	24.3	8.1	67.7
Safety															
Autism	1.9	14.3	83.7	6.9	32.7	60.4	5.6	39.4	55.0	8.1	45.4	46.5	5.6	36.2	58.2
Mercury	1.5	7.7	90.8	5.4	29.0	65.6	5.8	34.8	59.4	8.8	44.8	46.4	5.6	32.0	62.4
ChildSideEffects	4.8	2.4	92.8	8.4	6.1	85.5	9.5	7.6	82.9	14.3	16.1	69.6	9.5	8.0	82.5
ChildSafety	1.4	1.4	97.1	6.1	4.6	89.4	4.3	4.2	91.5	8.0	8.0	83.9	4.6	4.4	91.0
FluSideEffects	8.0	3.9	88.2	17.5	8.7	73.8	27.7	11.4	60.9	38.5	19.5	42.1	25.7	11.3	63.0
FluSafety	4.6	3.4	92.0	11.1	5.4	83.5	20.1	12.2	67.7	31.8	17.4	50.8	18.9	11.2	70.0
Trust															
QuestionDoctors	29.4	13.7	56.9	36.5	14.2	49.2	43.8	17.4	38.9	52.8	19.4	27.8	42.5	16.9	40.6
PatientsBest	0.5	1.0	98.5	3.7	1.5	94.6	3.9	5.0	91.1	7.0	9.7	83.3	3.9	4.9	91.3
DoctorsAuthority	4.2	10.5	85.3	13.6	17.5	68.9	14.1	19.7	66.2	17.8	33.5	48.8	13.2	20.3	66.5
HealthDecisions	19.2	13.1	67.6	37.2	14.9	47.9	36.4	18.3	45.2	41.6	20.8	27.6	34.9	17.7	47.4

% Neg = Percentage of HCWs who answered *strongly disagree* or *disagree* (*agree* or *strongly agree* on reverse-scored items). % Mid = Percentage of HCWs who answered *neither agree nor disagree*. % Pos = Percentage of HCWs who answered *agree* or *strongly disagree* (*strongly disagree* or *disagree* on reverse-scored items).

The HCWs’ responses to the questions on whether they accepted childhood vaccines for their own children and influenza vaccines for themselves, as well as whether they recommend childhood and influenza vaccines to hesitant patients, are shown in Table 4. Of the HCWs who reported having children (*n* = 2234), the great majority had never hesitated in a vaccination decision, or postponed or rejected a vaccination for their child (81.6%). The proportion of HCWs who had received the influenza vaccine the previous season was also high (86.2%). Most of the HCWs whose duties involved discussing and/or administering vaccines on a weekly basis (*n* = 792 for childhood vaccines; *n* = 802 for influenza vaccines) indicated that they guide vaccine-hesitant patients towards accepting the childhood and influenza vaccines (85.7% and 73.6%, respectively).

**Table 4:** Descriptive statistics of the HCWs’ own vaccination decisions and recommendation behavior.

	Doctors		Head nurses		Nurses		Practical nurses		Total	
Variable	*n*	%	*n*	%	*n*	%	*n*	%	*n*	%
Vaccines for own children										
No hesitation/postponing/rejection	289	88.1	187	81.3	1048	79.4	299	84.0	1823	81.6
Hesitated	23	7.0	27	11.7	195	14.8	46	12.9	291	13.0
Postponed	11	3.4	16	7.0	96	7.3	17	4.8	140	6.3
Rejected	9	2.7	9	3.9	66	5.0	7	2.0	91	4.1
Medical reasons	11	3.4	12	5.2	49	3.7	9	2.5	81	3.6
Total	328	100.0	230	100.0	1320	100.0	356	100.0	2234	100.0
Own influenza vaccination										
No	22	5.3	15	5.7	225	12.4	136	30.4	398	13.5
Yes	392	94.7	247	93.9	1584	87.3	311	69.4	2534	86.2
Medical reasons	0	0.0	1	0.4	5	0.3	1	0.2	7	0.2
Total	414	100.0	263	100.0	1814	100.0	448	100.0	2939	100.0
Childhood vaccine recommendation										
Guide parent towards not vaccinating	0	0.0	0	0.0	2	0.4	2	3.4	4	0.5
Do not guide parent	4	2.9	10	20.4	82	15.0	13	22.4	109	13.8
Guide parent towards vaccinating	136	97.1	39	79.6	461	84.6	43	74.1	679	85.7
Total	140	100.0	49	100.0	545	100.0	58	100.0	792	100.0
Influenza vaccine recommendation										
Guide patient towards not vaccinating	0	0.0	1	2.0	1	0.2	1	1.7	3	0.4
Do not guide patient	12	8.3	9	17.6	167	30.4	21	36.2	209	26.1
Guide patient towards vaccinating	132	91.7	41	80.4	381	69.4	36	62.1	590	73.6
Total	144	100.0	51	100.0	549	100.0	58	100.0	802	100.0

The rows Hesitated, Postponed, and Rejected are not mutually exclusive, as a respondent may have answered yes to all three questions.

### Main analyses

#### Evaluation of the measurement models

The fit of the three factors Benefit, Safety, and Trust, when analyzed in separate models, can be seen in S3 Table. In the three-factor model, three error correlations were re-specified as freely estimated based on investigation of modification indices and theoretical considerations. The fit indices of the final measurement model indicated adequate fit, *χ*^2^(146) = 2328.94, CFI = .945, TLI = .936, RMSEA = .071, SRMR = .057, and all indicators had significant standardized factor loadings above .40. However, the correlation between the factors Benefit and Safety was very strong (*r* = .93). We therefore decided to collapse the two factors into one factor labeled BenefitSafety. The two-factor model also showed adequate fit, *χ*^2^(148) = 2424.56, CFI = .943, TLI = .934, RMSEA = .072, SRMR = .058. Again, all indicators had standardized loadings above .40 (Table 5). Statistical comparison of the three-factor and the two-factor models showed that the three-factor solution was a significantly better fit to the data, *χ*^2^_diff_(2) = 93.83, *p* < .001. However, due to the strong correlation between Benefit and Safety that indicated low discriminant validity between the factors, as well as the small difference in fit, we decided to retain the two-factor model. The correlation between the two remaining factors, BenefitSafety and Trust, was .74.

**Table 5:** Factor loadings and variances from confirmatory factor analysis of the two-factor model.

		Unstandardized			Standardized		
Factor	Parameter	Estimate	*SE*		Estimate	*SE*	*R*^2^
Factor loadings							
BenefitSafety	HerdImmunity	1.00	−		0.65	0.03	0.42
	Immunized	0.88	0.04		0.57	0.01	0.33
	NotCommon	0.62	0.03		0.40	0.02	0.16
	ChildProtection	0.97	0.04		0.63	0.02	0.40
	ChildSerious	0.72	0.04		0.47	0.02	0.22
	ChildNecessary	1.07	0.05		0.69	0.02	0.48
	FluProtection	1.02	0.05		0.66	0.01	0.44
	FluSerious	1.07	0.05		0.70	0.02	0.49
	FluNecessary	1.04	0.04		0.68	0.01	0.46
	Autism	0.86	0.04		0.56	0.02	0.31
	Mercury	1.00	0.04		0.65	0.01	0.43
	ChildSideEffects	0.93	0.04		0.60	0.02	0.36
	ChildSafety	1.01	0.04		0.66	0.01	0.43
	FluSideEffects	1.25	0.05		0.81	0.01	0.66
	FluSafety	1.25	0.05		0.81	0.01	0.66
Trust	QuestionDoctors	1.00	−		0.51	0.02	0.26
	PatientsBest	1.47	0.07		0.75	0.02	0.57
	DoctorsAuthority	1.20	0.06		0.62	0.02	0.38
	HealthDecisions	0.89	0.05		0.46	0.02	0.21
Factor variances							
BenefitSafety		0.42	0.03		1.00	−	−
Trust		0.26	0.02		1.00	−	−

Residual correlations included Autism and Mercury (*r* = .60, *p* < .001), ChildProtection and ChildSafety (*r* = .67, *p* < .001), and FluProtection and FluSafety (*r* = .44, *p* < .001).

### Structural regression models

After having confirmed an acceptable measurement model, we fitted the four SR models to the data. All four SR models included the two latent variables BenefitSafety and Trust as predictors, as well as one of the four outcome variables that was regressed on the two latent factors. With 210 elements in the input matrix and 45 free parameters, each model was identified with *df* = 165.

The model for childhood vaccination decisions was conducted with only those 2234 (75.4%) respondents who reported having children. The SR model, *χ*^2^(165) = 1820.47, CFI = .944, TLI = .935, RMSEA = .067, SRMR = .057, showed a significant negative association between BenefitSafety and vaccination decisions concerning own children, *β* = -.38, 95% CI [-.51, -.25], *SE* = 0.07, *Z* = 5.80, *p* < .001. This indicated that HCWs who perceived vaccines as less beneficial and safe, more often reported hesitation, postponing, or rejection in connection to vaccination decisions concerning their children. However, there was no significant association between Trust and childhood vaccination decisions concerning the HCWs’ own children, *β* = -.03, 95% CI [-.17, .11], *SE* = 0.07, *Z* = 0.47, *p* = .636.

The model concerning influenza vaccination behavior was fit to the whole sample of HCWs (*N* = 2962). In this SR model, *χ*^2^(165) = 2630.38, CFI = .941, TLI = .932, RMSEA = .071, SRMR = .062, BenefitSafety was significantly related to whether the HCWs had received the influenza vaccine the previous season or not, *β* = -.79, 95% CI [-.89, -.69], *SE* = 0.05, *Z* = 16.16, *p* < .001. This indicated that individuals who perceived vaccines as beneficial and safe were more likely to have taken the influenza vaccine. Furthermore, Trust showed a statistically significant association with influenza vaccination behavior, *β* = .12, 95% CI [.01, .24], *SE* = 0.06, *Z* = 2.04, *p* = .041, indicating that, contrary to our hypothesis, the HCWs with lower trust were more likely to have taken the influenza vaccine. However, the association was very weak, and therefore, we will not consider this result further.

The models including the outcome variables related to recommendation behavior were fitted using the sample of HCWs who reported that their work duties involved discussing and/or administering vaccines on a weekly basis (*n* = 810; 27.3%). The SR model on childhood vaccine recommendation, *χ*^2^(165) = 577.80, CFI = .950, TLI = .942, RMSEA = .056, SRMR = .060, revealed a significant negative relationship between BenefitSafety and recommending vaccines, *β* = -.41, 95% CI [.23, .59], *SE* = 0.09, *Z* = 4.56, *p* < .001. Thus, the results suggested that HCWs who perceived vaccines as beneficial and safe more often reported that they recommend vaccines to hesitant patients. The same pattern was found in the second recommendation-related SR model, *χ*^2^(165) = 658.20, CFI = .943, TLI = .935, RMSEA = .061, SRMR = .065, where BenefitSafety was negatively related to recommendation behavior concerning the influenza vaccine, *β* = -.65, 95% CI [.53, .78], *SE* = 0.06, *Z* = 10.13, *p* < .001. By contrast, Trust was not a significant predictor of the HCWs’ likelihood of recommending childhood vaccinations, *β* = -.10, 95% CI [-.10, .29], *SE* = 0.10, *Z* = 0.94, *p* = .349, or influenza vaccinations, *β* = -.01, 95% CI [-.15, .17], *SE* = 0.08, *Z* = 0.11, *p* = .916, to their patients in neither of the models.

We also fitted each SR model to the data excluding the practical nurses to investigate whether the pattern of associations would differ when including only individuals who have the right to administer vaccines. Practical nurses do not have the right to administer vaccines in Finland. The results from these control analyses were in line with the abovementioned findings for all other associations except for Trust, which was not significantly associated with the HCWs’ own influenza vaccination status (see, S4 Table).

#### Follow-up analyses for professional groups

Because the results from the abovementioned SR models showed that BenefitSafety predicted both the HCWs’ own vaccination behavior and recommend vaccines to hesitant patients, we investigated whether the professional groups differed in their perceptions of vaccines as beneficial and safe. In this follow-up SR analysis, BenefitSafety was set as the outcome variable, while the professions were compared using repeated contrasts according to the level of education (practical nurses vs. nurses; nurses vs. head nurses; head nurses vs. doctors). The analysis was run using the full sample of HCWs (*n* = 2962). The model, *χ*^2^(129) = 1832.25, CFI = .926, TLI = .940, RMSEA = .067, SRMR = .070, revealed significant differences for all comparisons. Practical nurses perceived vaccines less beneficial and safe than nurses did, *β* = -.16, 95% CI [-.19, -.12], *SE* = 0.02, *Z* = 8.82, *p* < .001, nurses considered vaccines less beneficial and safe than head nurses did, *β* = -.13, 95% CI [-.18, -.08], *SE* = 0.03, *Z* = 4.63, *p* < .001, and head nurses perceived vaccines less beneficial and safe than doctors did, *β* = -.23, 95% CI [-.28, -.18], *SE* = 0.03, *Z* = 8.79, *p* < .001.

**Fig 1:**
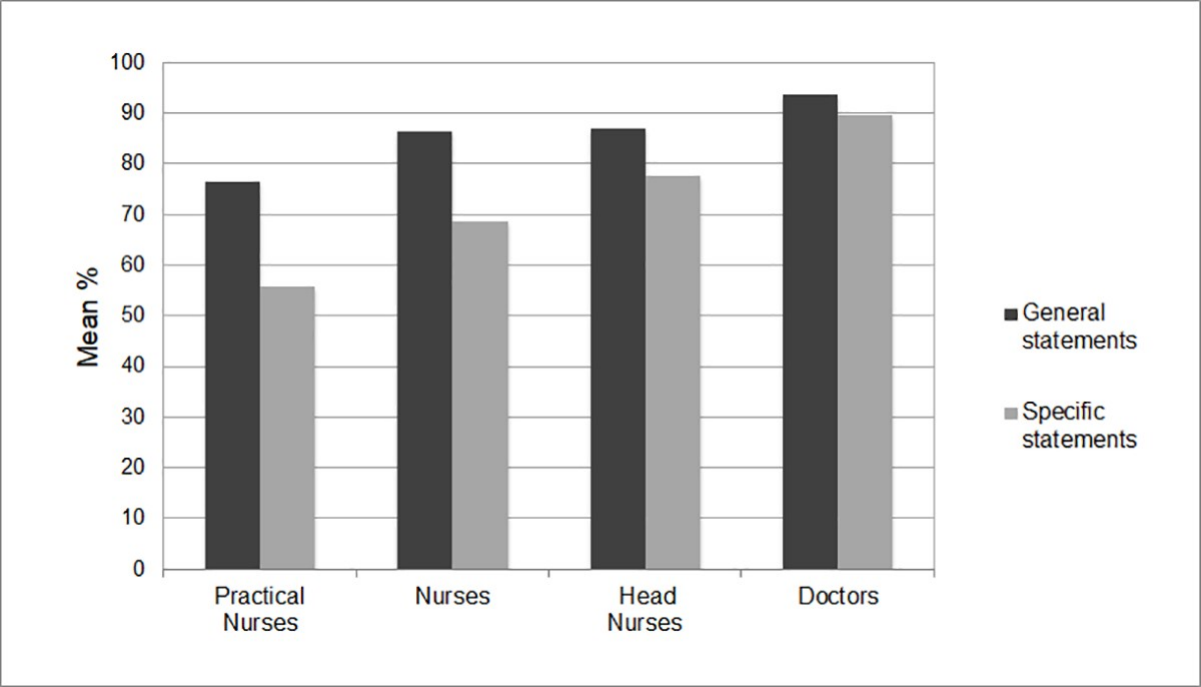
Proportion of HCWs by profession agreeing with general and specific statements (disagreeing with reversed statements).

Fig 1 displays the HCWs’ attitudes related to the statements included in the factor BenefitSafety, divided by whether the statements queried general or specific knowledge. The bars represent the percentage of HCWs that report positive attitudes, averaged over the items. An inspection of the mean percentages for each profession separately, indicated that all professions agreed more with general statements compared with specific ones. However, the difference between the proportions of individuals agreeing with general and specific statements was smallest for doctors and increased as the educational level of the HCWs decreased.

## Discussion

In the present study we investigated if Finnish HCWs’ perceptions of the benefits and safety of vaccines, and trust in health professionals, is related to their decisions to accept vaccinations for themselves and their children, and to their willingness to recommend vaccines to vaccine-hesitant patients. The majority of the HCWs perceived vaccines to be beneficial and safe, and trusted health professionals. Nonetheless, a notable share of the HCWs questioned the benefits and safety of vaccines, and expressed distrust in the professional competence and intentions of health professionals. We review and explore the implications of the core findings.

### Own vaccination behavior

Most HCWs reported accepting all childhood vaccinations for their children (95.9%). However, 13.0% reported that they had hesitated in a vaccination decision, 6.3% that they had postponed a vaccination, and 4.1% that they had rejected a vaccination for their children. These results are in line with previous research suggesting that hesitancy towards vaccines exists among HCWs as well [21–23]. The uptake of the influenza vaccine was high in the present sample of HCWs (86.2% vaccinated) compared to uptake rates reported in other countries [28]. This might be due to the new Infectious Diseases Act [36] and the work-related consequences the Finnish HCWs might face if they do not take the vaccine against influenza. However, data on the uptake of influenza vaccines among Finnish HCWs at Turku University Hospital (the personnel from this hospital was not included in the present study) collected before the act came into effect (influenza season 2015–2016) indicate a vaccination rate of 63% [42]. In European countries where influenza vaccinations for HCWs are not mandatory, uptake rates are typically lower than 30% [28].

The results from the present study further showed that HCWs’ perception and knowledge about the benefits and safety of vaccines were related to their own vaccination behavior, both in terms of allowing their children to get vaccinated in accordance with the national vaccination program, and in terms of accepting the vaccine against seasonal influenza for themselves. In agreement with previous research in the general population, as well as in HCW samples [12–14,20,28,29,31], respondents with more positive attitudes towards vaccines were less likely to have hesitated, postponed or rejected vaccinations for their children, and were more likely to have accepted the influenza vaccine during the previous influenza season. The HCWs’ trust in health professionals was not directly associated with the decision to vaccinate their children, when their perceptions of the benefits and safety of the vaccines were taken into account (see below).

### Recommendation behavior

Very few HCWs reported that they guide hesitant patients towards not vaccinating (0.5% and 0.4% for childhood and influenza vaccination respectively). However, 13.8% reported that they do not guide the patient in any direction when the hesitancy concerns the childhood vaccines. This figure was approximately twice as high (26.1%) when the patients’ hesitancy concerned the influenza vaccine. The fact that a non-negligible proportion of the HCWs do not guide vaccine-hesitant patients towards taking the vaccine is worrying, as a lack of recommendation from health professionals is reported by laypeople as a reason for not taking the vaccine [8,12,18–20]. In an attempt to clarify why some HCWs do not guide their patients in immunization decisions, we investigated whether recommendation behavior was associated with their own attitudes towards vaccines and other health professionals. This was found to be the case for vaccine attitudes, as HCWs with more positive views on vaccine benefits and safety were also more likely to guide hesitant patients towards accepting childhood and influenza vaccines. This relationship between vaccine attitudes and recommendation behavior has been observed in previous research [21,26,27]. Trust in health professionals was not, however, directly related to recommendation behavior.

### Trust in health professionals

As previously mentioned, and contrary to our hypothesis, the HCWs’ trust in health professionals was not directly associated with the HCWs’ own vaccination behavior or their willingness to recommend vaccines to vaccine-hesitant patients. This finding stands in apparent conflict with the results from previous studies suggesting that HCWs with a higher trust in the health system are also more likely to take the influenza vaccine and to recommend vaccines to patients [27,31]. The main difference between the present study and the two previous ones investigating this association is the operationalization of trust. In the present study, the statements designed to measure trust concerned the HCWs’ trust in the professional competence and intentions of doctors and health professionals in general. In previous studies, on the other hand, trust was operationalized as trust in health authorities, scientific sources, and experts [27], or professional recommendations [31], concerning vaccine-related circumstances in particular. The difference between results might, hence, be due to the different aspects of trust that were measured. Another possible reason for the discrepancy in results relates to how the data were analyzed. In the present study, the correlation between the HCWs’ perception of the benefits and safety of vaccines and their trust in health professionals was strong (*r* = .74), indicating that trust is strongly related to vaccine attitudes. In the multivariate regression model, however, where the two predictors (BenefitSafety and Trust) compete with one another, perceptions of the benefit and safety of vaccines showed a unique association with the outcome measures, while there was no unique relation of trust in health professionals on the outcome measures. In the study by Učakar et al [31], other variables were not controlled for. The analyses in the study by Collange et al [27] included several predictors, of which some were not investigated in the present study (such as personal and professional characteristics). Furthermore, cultural differences between the investigated populations might explain the conflicting results, as trust issues are contextual and vary between countries [24]. Also, the samples utilized by the abovementioned studies were more homogenous, including doctors only, while here, four different professional groups were included in the analyses.

### Profession

As the HCWs’ perception and knowledge about the benefits and safety of vaccines predicted both their own vaccination behavior and their willingness to recommend vaccines to hesitant patients, we conducted follow-up analyses to investigate if the levels of confidence differed between the various professional groups. Those analyses showed statistically significant differences between all professional groups, with doctors being most confident, followed by head nurses, nurses, and practical nurses. This finding suggested that the degree to which the HCWs’ perceptions were in line with scientific evidence, meaning that they perceived vaccines to be beneficial and safe, was positively related to their level of education. Also previous research has found that, in general, doctors have more positive attitudes towards vaccines than nurses [23]. Furthermore, the difference between professions seemed to be greater for statements that required specific knowledge (i.e., related to specific vaccines or diseases) than for more general statements (i.e., related to vaccines in general; Fig 1). In other words, the results indicated some uncertainty among HCWs when it comes to more specific knowledge about vaccines or the diseases they prevent. This was the case particularly for HCWs with lower levels of education. Taken together, these findings might indicate that confidence in the benefits and safety of vaccines among HCWs is related to the amount of medical training they have received. If this is the case, higher confidence may be achieved by providing HCWs with more vaccine-related education. The relationship between confidence and professional group can, however, also be explained by other factors, such as differences in professional identity or amount or type of vaccine-related work. This possible link between profession and vaccine confidence is an important question to be addressed in future research.

### Limitations

There are some important issues to take into account when interpreting the results of the present study. First, the results are based on self-reported attitudes and behaviors. Therefore, the responses might be affected by, for example, desirability bias or memory issues. Using official vaccination records might lead to more accurate measures of vaccination behavior in terms of uptake, and observing HCWs during their encounters with patients would yield more accurate and detailed information about their communication behavior. However, the methods utilized in the present study enabled the collection of information from a large sample of HCWs, which increases the generalizability of the results.

Second, the questionnaire employed was developed for the purpose of the present study and has not been validated in other samples. However, face validity of the questionnaire was assessed by experts in the field and factor analysis was utilized to evaluate the degree to which the questions loaded on the constructs and to handle measurement error.

Third, the data collection was cross-sectional and, hence causality cannot be inferred with certainty. There are, however, good reason to suppose that the confidence HCWs have in vaccines and healthcare, determines their vaccination and recommendation behavior, rather than the other way around.

Fourth, our study population did not include HCWs working at child health clinics. This limits the generalizability of our findings especially when it comes to the recommendation behavior related to childhood vaccines, as most childhood vaccinations in Finland are carried out at child health clinics by public health nurses. To what degree the present results apply also to HCWs working at child health clinics, is therefore an important topic for future studies.

### Conclusions

The results from the present study suggest that the majority of Finnish HCWs have high confidence in the benefits and safety of vaccines and show trust in other health professionals. However, low vaccination confidence was found among a non-negligible proportion of the HCWs. The results further showed that HCWs who perceived vaccines as less beneficial and safe were also less likely to have accepted vaccines for themselves and for their children, and were less willing to recommend vaccines to vaccine-hesitant patients. Trust in health professionals was not directly associated with the HCWs’ own vaccination decisions or willingness to recommend vaccines.

Confidence in evidence-based information on vaccinations seemed to be related to the level of education among the HCWs, as the degree of confidence increased along with the educational level. This was the case in particular for statements requiring knowledge about specific vaccines or diseases. Further research should examine whether vaccination confidence can be increased by more vaccine-related education or training. Ensuring that HCWs have high confidence in vaccines may be important for maintaining high vaccine uptake in the general population, as assurance by HCWs is reported by laypeople as a key factor in their decision to get vaccinated.

## Supporting information

S1 Appendix Complete Questionnaire. (DOCX)

S1 Table Amount of missing responses per variable included in the SR models. (DOCX)

S2 Table Descriptive information on vaccination confidence in HCWs with the right to administer vaccines who have vaccine-related work on a weekly basis. (DOCX)

S3 Table Fit statistics of the one-factor models. (DOCX)

S4 Table Fit statistics and probit regression coefficients of the SR models for respondents with the right to administer vaccines. (DOCX)
