# Supplementary material for: The association between vaccination confidence, vaccination behavior, and willingness to recommend vaccines among Finnish healthcare workers
Source: PLoS One. 2019 Oct 31;14(10):e0224330. doi: 10.1371/journal.pone.0224330 (PMC6822763; doi:10.1371/journal.pone.0224330)
Supplement: S1 Appendix — (DOCX) [file pone.0224330.s001.docx]

**S1 Appendix. Complete Questionnaire.**

* = mandatory question (the participants can skip questions without *)

(IF ...) = whether the question is presented or not is dependent on the participant’s answers to previous questions (questions without IFs are presented to all participants)

1) Informed consent

2) Please read the statements below and indicate how much you agree with the statements by choosing a response alternative on the scale.

|  | Strongly disagree | Partially disagree | Neither agree nor disagree | Partially agree | Strongly agree |
| --- | --- | --- | --- | --- | --- |
| Vaccinating healthy children helps to protect others by stopping the spread of disease. | 1 | 2 | 3 | 4 | 5 |
| It is better to be immunized trough the disease than through the vaccine. | 1 | 2 | 3 | 4 | 5 |
| Vaccines can cause autism. | 1 | 2 | 3 | 4 | 5 |
| Vaccines contain dangerous quantities of mercury. | 1 | 2 | 3 | 4 | 5 |
| Children need vaccines for diseases that are not common anymore. | 1 | 2 | 3 | 4 | 5 |
| If you travel to countries where diseases such as hepatitis A and B, yellow fever, or Japanese encephalitis are common, it is important to take the vaccines that should protect against them. | 1 | 2 | 3 | 4 | 5 |

The term “childhood vaccines” refer to the vaccines included in the national vaccination programme in Finland for children six years old and younger. This vaccination programme includes:

- the rotavirus vaccine
- the pneumococcal conjugate vaccine (PCV; against meningitis, pneumonia, sepsis, and ear infection)
- the DTaP-IPV-Hib vaccine (the ”5-in-1 vaccine”; against diphtheria, tetanus, pertussis, polio, and Hib diseases such as meningitis, epiglottitis, and sepsis)
- the MMR vaccine (against measles, mumps, and rubella)
- the DtaP-IPV vaccine (the ”4-in-1 vaccine”; against diphtheria, tetanus, pertussis, and polio)
- the chickenpox vaccine

Thus, the questions do NOT concern the HPV-vaccine (against cervical cancer).

3) Please read the statements below and indicate how much you agree with the statements by choosing a response alternative on the scale.

|  | Strongly disagree | Partially disagree | Neither agree nor disagree | Partially agree | Strongly agree |
| --- | --- | --- | --- | --- | --- |
| The risk of side effects outweighs the protective benefits of the **childhood vaccines**. | 1 | 2 | 3 | 4 | 5 |
| **Childhood vaccines** are safe. | 1 | 2 | 3 | 4 | 5 |
| **Childhood vaccines** are effective in protecting against diseases. | 1 | 2 | 3 | 4 | 5 |
| Measles is a very serious disease. | 1 | 2 | 3 | 4 | 5 |
| A good hygiene will make measles disappear from society – the vaccine is not necessary. | 1 | 2 | 3 | 4 | 5 |
| I think it is important that healthcare professionals recommend **childhood vaccines** to their patients (as long as they do not have a medical contraindication). | 1 | 2 | 3 | 4 | 5 |

The term “influenza vaccines” refers to the seasonal vaccines for influenza (including swine flu).

4) Please read the statements below and indicate how much you agree with the statements by choosing a response alternative on the scale.

|  | Strongly disagree | Partially disagree | Neither agree nor disagree | Partially agree | Strongly agree |
| --- | --- | --- | --- | --- | --- |
| The risk of side effects outweighs the protective benefits of the **influenza vaccines**. | 1 | 2 | 3 | 4 | 5 |
| The **influenza vaccines** are safe. | 1 | 2 | 3 | 4 | 5 |
| The **influenza vaccines** are effective in preventing the disease. | 1 | 2 | 3 | 4 | 5 |
| It is not worth getting the **influenza vaccine**, as the influenza symptoms are not serious. | 1 | 2 | 3 | 4 | 5 |
| Good hand hygiene and other preventive efforts are enough for avoiding the **influenza** even without vaccination. | 1 | 2 | 3 | 4 | 5 |
| I think it is important that healthcare professionals recommend **influenza vaccines** to their patients (as long as they do not have a medical contraindication). | 1 | 2 | 3 | 4 | 5 |
| I think it is important that healthcare professionals with patient work get vaccinated against **influenza** (as long as they do not have a medical contraindication). | 1 | 2 | 3 | 4 | 5 |

5) Please read the statements below and indicate how much you agree with the statements by choosing a response alternative on the scale.

|  | Strongly disagree | Partially disagree | Neither agree nor disagree | Partially agree | Strongly agree |
| --- | --- | --- | --- | --- | --- |
| I trust the information I receive from healthcare authorities about vaccines. | 1 | 2 | 3 | 4 | 5 |
| Health care authorities would not recommend vaccines that are unsafe. | 1 | 2 | 3 | 4 | 5 |
| I think it is good that patients/parents question the doctors’ ability to make correct diagnoses. | 1 | 2 | 3 | 4 | 5 |
| When healthcare professionals make medical decisions, they have the patients’ best interest in mind. | 1 | 2 | 3 | 4 | 5 |
| Doctors are too authoritative towards their patients. | 1 | 2 | 3 | 4 | 5 |
| Parents should leave the decisions that concern their children’s health in the healthcare professionals’ hands. | 1 | 2 | 3 | 4 | 5 |

6) *Do your duties include meeting and treating patients?

1. Yes

2. No

7) Approximately how many patients do you see per week? (IF 6 = Yes)

8) *How many times per week do your contact with patients concern vaccinations (e.g., discussion, administration)? (IF 6 = Yes)

1. 0

2. 1-3

3. 4-10

4. More than 10

9) Please read the statements below and indicate how much you agree with the statements by choosing a response alternative on the scale. (IF 8 = 1-3 OR 4-10 OR More than 10)

|  | Strongly disagree | Partially disagree | Neither agree nor disagree | Partially agree | Strongly agree |
| --- | --- | --- | --- | --- | --- |
| I feel I have enough information about vaccines to be able to answer my patients’ questions. | 1 | 2 | 3 | 4 | 5 |
| I see it as my obligation to recommend that the patient gets vaccinated. | 1 | 2 | 3 | 4 | 5 |
| I gladly discuss vaccines with my patients. | 1 | 2 | 3 | 4 | 5 |

10) If a patient is hesitant towards vaccines, it makes me feel… (IF 8 = 1-3 OR 4-10 OR More than 10)

|  | Not at all | Very little | Somewhat | A lot | Very much |
| --- | --- | --- | --- | --- | --- |
| ... angry. | 1 | 2 | 3 | 4 | 5 |
| ... irritated. | 1 | 2 | 3 | 4 | 5 |
| ... disappointed. | 1 | 2 | 3 | 4 | 5 |
| ... indifferent. | 1 | 2 | 3 | 4 | 5 |
| ... respect. | 1 | 2 | 3 | 4 | 5 |
| ... content. | 1 | 2 | 3 | 4 | 5 |
| ... happy. | 1 | 2 | 3 | 4 | 5 |

11) In percent, how many of your patients do you estimate are hesitant towards (e.g., express concern for side effects, consider not vaccinating) or oppose vaccines? (IF 8 = 1-3 OR 4-10 OR More than 10)

___ %

12) How do you proceed if a parent is unsure about a vaccination decision concerning the **childhood vaccines** (and the child does not have any medical contraindications)? (IF 8 = 1-3 OR 4-10 OR More than 10)

1. I try to guide the parent towards letting the child get vaccinated

2. I try to guide the parent towards *not* letting the child get vaccinated

3. I do not try to guide the parent in any direction

13) How do you proceed if a patient is unsure about a vaccination decision concerning an **influenza vaccine** (and the patient does not have any medical contraindications)? (IF 8 = 1-3 OR 4-10 OR More than 10)

1. I try to guide the patient towards vaccinating

2. I try to guide the patient towards *not* vaccinating

3. I do not try to guide the patient in any direction

14) Have you participated in the vaccination course arranged by the National Institute of Health and Welfare (THL) and Metropolia University of Applied Sciences? (IF 6 = Yes)

1. No

2. Yes

15) What year did you last participate in the vaccination course by THL and Metropolia? (IF 14 = Yes)

16) Did you take the last **influenza vaccine** (season 2017-2018)?

1. No

2. Yes

17) Will you take the **influenza vaccine** next season (season 2018-2019)?

1. No

2. Yes

3. I don’t know

18) Please state in the list below on what grounds you made the choice not to take the influenza vaccine or on what grounds you are hesitating. You can mark several alternatives if you wish. (IF 16 = No, OR 17 = No OR I don’t know)

□ Lack of time

□ Fear of needles

□ Religious convictions

□ Ethical convictions

□ Other convictions

□ I do/did not think that the vaccine was effective

□ I do/did not think that influenza is a serious disease

□ I do/did not think that the vaccine was safe because of its side effects

□ I think/thought that other preventive efforts are more effective

□ I deem/deemed it unlikely that I will fall ill with the influenza

□ I have had bad experiences or physical reactions at previous vaccinations

□ I do/did not know where to get good and reliable information about influenza vaccines

□ Other reasons, please specify:

19) *Do you have children?

1. No

2. Yes

20) Have you ever hesitated to letting your child(ren) receive any of the **childhood vaccines**? (IF 19 = Yes)

1. No

2. Yes

21) Have you ever postponed a vaccination for your child(ren) with any of the **childhood vaccines**? (IF 19 = Yes)

1. No

2. Yes

22) Have you ever decided not to let your child(ren) receive any of the **childhood vaccines**? (IF 19 = Yes)

1. No

2. Yes

23) Will you reject vaccination with any of the **childhood vaccines** for your child(ren) in the future? (IF 19 = Yes)

1. No

2. Yes

3. I don’t know

4. My child(ren) already have all the childhood vaccines

24) Please state in the list below on what grounds you have hesitated to vaccinate or postponed/rejected vaccination of you child(ren), or on what grounds you plan to reject vaccination in the future (or don’t know). You can mark several alternatives if you wish. (IF 20 = Yes OR 21 = Yes OR 22 = Yes OR 23 = Yes OR I don’t know)

□ Lack of time

□ Fear of needles

□ Religious convictions

□ Ethical convictions

□ Other convictions

□ I do/did not think that the vaccine was effective

□ I do/did not think that disease the vaccine is intended for is serious

□ I do/did not think that the vaccine was safe because of its side effects

□ I think/thought that other preventive efforts are more effective

□ I deem/deemed it unlikely that my child will fall ill with the disease the vaccine is intended for as others are vaccinated

□ I or my child have had bad experiences or physical reactions at previous vaccinations

□ I do/did not know where to get reliable information about vaccines

□ Other reasons, please specify:

25) Please read the statements below and indicate how much you agree with the statements by choosing a response alternative on the scale.

|  | Strongly disagree | Partially disagree | Neither agree nor disagree | Partially agree | Strongly agree |
| --- | --- | --- | --- | --- | --- |
| The media generally cover vaccination-related issues in a fair and reasonable manner. | 1 | 2 | 3 | 4 | 5 |
| The media often engage in ‘false balance’ by inviting anti-vaccination activists to counter messages from experts. | 1 | 2 | 3 | 4 | 5 |
| The media should focus on scientific expert opinions only when reporting on vaccination-related issues. | 1 | 2 | 3 | 4 | 5 |
| The media should provide a full spectrum of views on vaccination-related issues, even if that includes non-scientific voices. | 1 | 2 | 3 | 4 | 5 |

26) How often do you encounter vaccine-related reporting in media? Choose the alternative that fits best.

1. Never or very rarely

2. 1-3 times a month

3. About one time a week

4. Several times per week
