# Supplementary material for: The association between vaccination confidence, vaccination behavior, and willingness to recommend vaccines among Finnish healthcare workers
Source: PLoS One. 2019 Oct 31;14(10):e0224330. doi: 10.1371/journal.pone.0224330 (PMC6822763; doi:10.1371/journal.pone.0224330)
Supplement: S1 Table — (DOCX) [file pone.0224330.s002.docx]

**S1 Table. Amount of missing responses per variable included in the SR models.**

| Variable | *N* respondents | *n* missing | *%* missing |
| --- | --- | --- | --- |
| HerdImmunity | 2962 | 14 | 0.5 |
| Immunized | 2962 | 4 | 0.1 |
| NotCommon | 2962 | 34 | 1.1 |
| ChildProtection | 2962 | 35 | 1.2 |
| ChildSerious | 2962 | 51 | 1.7 |
| ChildNecessary | 2962 | 24 | 0.8 |
| FluProtection | 2962 | 56 | 1.9 |
| FluSerious | 2962 | 41 | 1.4 |
| FluNecessary | 2962 | 22 | 0.7 |
| Autism | 2962 | 19 | 0.6 |
| Mercury | 2962 | 38 | 1.3 |
| ChildSideEffects | 2962 | 9 | 0.3 |
| ChildSafety | 2962 | 10 | 0.3 |
| FluSideEffects | 2962 | 17 | 0.6 |
| FluSafety | 2962 | 22 | 0.7 |
| QuestionDoctors | 2962 | 32 | 1.1 |
| PatientsBest | 2962 | 38 | 1.3 |
| DoctorsAuthority | 2962 | 31 | 1.0 |
| HealthDecisions | 2962 | 17 | 0.6 |
| Childhood vaccine hesitancy | 2234 | 1 | 0.0 |
| Flu vaccine status | 2962 | 23 | 0.8 |
| Childhood vaccine communication | 810 | 18 | 2.2 |
| Flu vaccine communication | 810 | 8 | 1.0 |
