# Supplementary material for: The association between vaccination confidence, vaccination behavior, and willingness to recommend vaccines among Finnish healthcare workers
Source: PLoS One. 2019 Oct 31;14(10):e0224330. doi: 10.1371/journal.pone.0224330 (PMC6822763; doi:10.1371/journal.pone.0224330)
Supplement: S2 Table — (DOCX) [file pone.0224330.s003.docx]

**S2 Table. Descriptive information on vaccination confidence in HCWs with the right to administer vaccines who have vaccine-related work on a weekly basis (*n* = 751).**

|  | % Neg | % Mid | % Pos |
| --- | --- | --- | --- |
| Benefit | | | |
| HerdImmunity | 2.0 | 0.4 | 97.6 |
| Immunized | 13.6 | 11.6 | 74.8 |
| NotCommon | 7.9 | 7.9 | 84.1 |
| ChildProtection | 2.7 | 0.8 | 96.5 |
| ChildSerious | 4.4 | 4.4 | 91.3 |
| ChildNecessary | 0.9 | 2.1 | 96.9 |
| FluProtection | 31.2 | 10.8 | 58.0 |
| FluSerious | 4.6 | 6.5 | 88.9 |
| FluNecessary | 19.1 | 5.5 | 75.4 |
| Safety | | | |
| Autism | 4.1 | 28.2 | 67.6 |
| Mercury | 4.0 | 22.4 | 73.6 |
| ChildSideEffects | 8.7 | 4.0 | 87.3 |
| ChildSafety | 4.0 | 3.1 | 92.9 |
| FluSideEffects | 17.9 | 8.0 | 74.0 |
| FluSafety | 12.2 | 8.8 | 79.0 |
| Trust | | | |
| QuestionDoctors | 38.6 | 15.4 | 46.0 |
| PatientsBest | 1.8 | 3.3 | 95.0 |
| DoctorsAuthority | 9.6 | 15.1 | 75.4 |
| HealthDecisions | 33.2 | 16.0 | 50.8 |

% Neg = Percentage of HCWs who answered strongly disagree or disagree (agree or strongly agree on reversed-score items). % Mid = Percentage of HCWs who answered neither agree nor disagree. % Pos = Percentage of HCWs who answered agree or strongly disagree (strongly disagree or disagree on reversed-score items).
