# Supplementary material for: The association between vaccination confidence, vaccination behavior, and willingness to recommend vaccines among Finnish healthcare workers
Source: PLoS One. 2019 Oct 31;14(10):e0224330. doi: 10.1371/journal.pone.0224330 (PMC6822763; doi:10.1371/journal.pone.0224330)
Supplement: S3 Table — (DOCX) [file pone.0224330.s004.docx]

**S3 Table. Fit statistics of the one-factor models.**

| Model | Chi^2^ | *df* | CFI | TLI | RMSEA | SRMR |
| --- | --- | --- | --- | --- | --- | --- |
| Benefit^a^ | 292.47 | 24 | .96 | .95 | .06 | .04 |
| Safety^b^ | 140.82 | 7 | .99 | .99 | .08 | .03 |
| Trust | 70.60 | 2 | .96 | .89 | .11 | .05 |
| Vaccine endorsement^c^ | 2025.79 | 86 | .95 | .93 | .09 | .07 |

WLSMV estimation with delta parameterization. Missing data handled by pair-wise deletion.

^a^Model including three residual correlations.

^b^Model including two residual correlations.

^c^Model including four residual correlations.
