# Supplementary material for: The association between vaccination confidence, vaccination behavior, and willingness to recommend vaccines among Finnish healthcare workers
Source: PLoS One. 2019 Oct 31;14(10):e0224330. doi: 10.1371/journal.pone.0224330 (PMC6822763; doi:10.1371/journal.pone.0224330)
Supplement: S4 Table — (DOCX) [file pone.0224330.s005.docx]

**S4 Table. Fit statistics and probit regression coefficients of the SR models for respondents with the right to administer vaccines.**

| Outcome variable | *χ*^2a^ | CFI | TLI | RMSEA | SRMR | *β* | 95% *CI*  [LL, UL] | *SE* | *Z* | *p* |
| --- | --- | --- | --- | --- | --- | --- | --- | --- | --- | --- |
| Vaccines for own children | 1300.00 | .95 | .94 | .06 | .06 |  |  |  |  |  |
| Benefit/Safety |  |  |  |  |  | -.43 | [-.57, -.30] | 0.07 | 6.34 | < .001 |
| Trust |  |  |  |  |  | .00 | [-.14, .15] | 0.08 | 0.04 | .967 |
| Own influenza vaccinations | 1873.41 | .95 | .94 | .06 | .06 |  |  |  |  |  |
| Benefit/Safety |  |  |  |  |  | -.76 | [-.86, -.65] | 0.06 | 13.76 | < .001 |
| Trust |  |  |  |  |  | .08 | [-.05, .21] | 0.07 | 1.21 | .225 |
| Childhood vaccine communication | 507.16 | .95 | .94 | .05 | .06 |  |  |  |  |  |
| Benefit/Safety |  |  |  |  |  | .39 | [.20, .58] | 0.10 | 4.00 | < .001 |
| Trust |  |  |  |  |  | .13 | [-.08, .34] | 0.11 | 1.19 | .234 |
| Influenza vaccine communication | 578.91 | .94 | .93 | .06 | .07 |  |  |  |  |  |
| Benefit/Safety |  |  |  |  |  | .61 | [.47, .74] | 0.07 | 8.86 | < .001 |
| Trust |  |  |  |  |  | .08 | [-.09, .24] | 0.09 | 0.88 | 380 |

WLSMV estimation with delta parameterization. Missing data handled by pair-wise deletion.

^a^*df* = 165.
